# Rehydration Compensation of Winter Wheat Is Mediated by Hormone Metabolism and De-Peroxidative Activities Under Field Conditions

**DOI:** 10.3389/fpls.2022.823846

**Published:** 2022-02-24

**Authors:** Xuejing Liu, Xiaodong Wang, Pan Liu, Xiaoyuan Bao, Xiaoyang Hou, Mingming Yang, Wenchao Zhen

**Affiliations:** ^1^State Key Laboratory of North China Crop Improvement and Regulation, Key Laboratory of Crop Growth Regulation of Hebei Province, College of Agronomy, Hebei Agricultural University, Baoding, China; ^2^College of Plant Protection, Hebei Agricultural University, Baoding, China; ^3^College of Agronomy, Northwest A&F University, Xianyang, China

**Keywords:** wheat, rehydration compensation, drought stress, hormone metabolism, transcriptomic, de-peroxidative stress

## Abstract

Water deficit and rehydration frequently occur during wheat cultivation. Previous investigations focused on the water deficit and many drought-responsive genes have been identified in winter wheat. However, the hormone-related metabolic responses and de-peroxidative activities associated with rehydration are largely unknown. In this study, leaves of two winter wheat cultivars, “Hengguan35” (HG, drought-tolerant cultivar) and “Shinong086” (SN, drought-sensitive cultivar), were used to investigate water deficit and the post-rehydration process. Rehydration significantly promoted wheat growth and postponed spike development. Quantifications of antioxidant enzymes, osmotic stress-related substances, and phytohormones revealed that rehydration alleviated the peroxidation and osmotic stress caused by water deficit in both cultivars. The wheat cultivar HG showed a better rehydration-compensation phenotype than SN. Phytohormones, including abscisic acid, gibberellin (GA), jasmonic acid (JA), and salicylic acid (SA), were detected using high-performance liquid chromatography and shown to be responsible for the rehydration process. A transcriptome analysis showed that differentially expressed genes related to rehydration were enriched in hormone metabolism- and de-peroxidative stress-related pathways. Suppression of genes associated with abscisic acid signaling transduction were much stronger in HG than in SN upon rehydration treatment. HG also kept a more balanced expression of genes involved in reactive oxygen species pathway than SN. In conclusion, we clarified the hormonal changes and transcriptional profiles of drought-resistant and -sensitive winter wheat cultivars in response to drought and rehydration, and we provided insights into the molecular processes involved in rehydration compensation.

## Introduction

Wheat is an important global staple crop vulnerable to climate conditions and its yield is associated with water supply during the cultivation season (Araya et al., 2019; Wang et al., 2021). Winter wheat relies on over-extracted groundwater to maintain a high yield, which causes severe ecological problems, including large-scale soil surface subsidence (Kong et al., 2016). Therefore, winter wheat production exacerbates water resource shortages, which is becoming increasingly serious (Huang et al., 2019). Rehydration compensation is a common phenomenon during crop cultivation. In certain developmental periods of crops, water limited irrigation triggers a compensatory promotion of growth after release of the drought stress, which simultaneously improves water use efficiency, yield, and quality of crops (Araya et al., 2019). An efficient rehydration compensation is dependent on a good balancing between “self-protection” of crops upon drought stress and “compensation” after rehydration treatment (Zhao et al., 2020). Consequently, it is crucial to clarify the physiological changes and molecular mechanisms associated with both water deficit and rehydration in wheat (Lopes et al., 2012; Song et al., 2017).

Morphological adaptations of crops are common features of rehydration compensation (Ahmad et al., 2015; Chen et al., 2020; Khadka et al., 2020). Compared with normal-watered plants, rehydration treatment at the re-greening stage of wheat can even elevate plant height, ear length, and flag leaf area (Baczek-Kwinta et al., 2006; Trujillo et al., 2013; Caldeira et al., 2014; Husen et al., 2014; Wang et al., 2017). Plant antioxidant metabolism is closely associated with water deficit and rehydration (Baczek-Kwinta et al., 2006; Furlan et al., 2016). Antioxidant enzymes are maintained at higher levels to balance the free radicals in plants to avoid damage from peroxidation under drought-stress conditions. During drought stress and rehydration, crops maintain a dynamic balance between the production and elimination of free radicals of ROS by regulating genes encoding antioxidant enzymes (He et al., 2012; Gupta et al., 2020), which has been evidenced by a series of studies in cotton, maize, and other crops (Koffler et al., 2014; Ashraf et al., 2015; Yi et al., 2016). For example, the activities of antioxidant enzymes superoxide dismutase (SOD) and peroxidase (POD) in maize leaves increased slowly upon drought stress but decreased dramatically after rehydration (Zheng et al., 2010; Ye et al., 2016). The degree of rehydration compensation is dependent on the tolerance of plant to drought stress (Zhang et al., 2018; Li X. R. et al., 2020). The association between osmotic stress regulatory ability and rehydration compensation have been evaluated in soybean and maize (Blum, 2017; Du et al., 2020). Genes regulating the production of proline (Pro), including pyrroline-5-carboxylate synthetase, harpin-encoding protein, and EF-Hand family protein, participate in plant tolerance to drought stress (Wang et al., 2013; Qiu et al., 2020; Anwar et al., 2021). The *Arabidopsis* C-repeat-binding factor gene (*AtCBF4*) is another key component in producing soluble sugars and enhancing the drought tolerance of plants (Zhang Z. et al., 2020). Water deficit triggers complex signal transduction pathways involved in hormone metabolism. The phytohormone changes result in plant physiological, ecological, and morphological responses against abiotic stresses. Upon drought stress, abscisic acid (ABA) accumulated in plant leaves and suppressed stomatal conduction, which eventually reduced the transpiration rate and prevented excessive water loss (Mega et al., 2019). The *Oryza sativa* Abscisic Stress-ripening 5 (*OsASR5*) gene plays an essential role in responses to drought stress by regulating ABA biosynthesis, promoting stomatal closure, and preventing drought stress-related protein inactivation (Li et al., 2017). Sajjad et al. (2021) found that a knock-out of cotton (*Gossypium hirsutum*) WUSCHEL-like homeobox 4 (*GhWOX4*) gene results in severely impaired vascular growth and drought tolerance. Several phytohormones, including auxin (AUX), indoleacetic acid (IAA), ABA and ethylene, are significantly induced in *Arabidopsis GhWOX4-*overexpression lines (Sajjad et al., 2021). During the rehydration, phytohormones also play crucial roles in the recovery of plant growth and long-term compensation (Truskina et al., 2021). To date, investigations have focused on the identification and characterization of drought-responsive genes in winter wheat, but little is known about the regulatory mechanisms of rehydration compensation. This study explored the hormonal changes and transcriptional profiles of drought-resistant and -sensitive winter wheat cultivars in response to rehydration. The physiological responses and molecular mechanisms of wheat plants during rehydration compensation were clarified.

## Materials and Methods

### Plant Material, Growth Conditions, Drought Stress, and Rehydration Treatment

**FIGURE 1:**
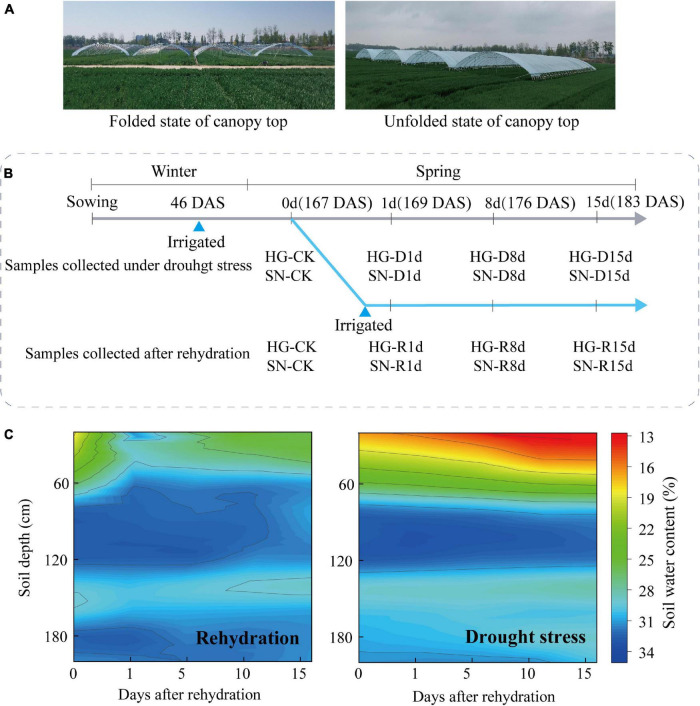
Schematic representation of rehydration experimental design. **(A)** Rain shelter for field experiments. **(B)** Timeline for rehydration treatment and sample collection. **(C)** Profile of the relative soil-water content (RSWC) during different rehydration stages. Scale bar represents soil moisture content in percentage. R, rehydrated plants; D, plants under continuous drought stress.

Drought-tolerant variety Hengguan35 (HG, sourced from Drought Agricultural Experiment Station of Hebei Academy of Agriculture and Forestry Sciences, Hebei, China) and drought-sensitive variety Shinong086 (SN, sourced from Hebei Dadi Seed Industry Co., Ltd., Hebei, China) were used in this study (Li D. et al., 2020). The seeds were sown in a field at the Shenzhou Experimental Station of Arid Crops Research Institute, Hebei Academy of Agriculture and Forestry Sciences (Hebei, China, 37.91°N, 115.71°E) on October 13, 2018. All winter wheat plants were grown under rain-proof shelters to prevent watering from the rain (Figure 1A). The area of each plot was 40 m^2^ (10 m × 4 m). On November 28, 2018, 3 m^3^ water was irrigated for each plot, as to ensure safety of overwinter. All wheat plants were subjected to continuous drought stress until the fourth-leaf stage in spring of 2019. A Trimer Pico 64 portable soil moisture meter (TDR, IMIKO, Germany) was used to measure the 0–200 cm layer soil moisture content (v/v) at every 20 cm soil depth. At the four-leaf stage of wheat plant [168 days after seed sowing (DAS)], and the soil water content of 0–60 cm layer was about 20%, two treatments were set up (Figure 1B). For Treatment 1 (rehydrated treatment), irrigation was applied, each plot was supplemented with 2.8 m^3^ water for irrigation until the water content of the 0–60 cm soil layer reached 80% of the field water-holding capacity. For Treatment 2 (continuous drought stress treatment), no irrigation was applied. Each treatment includes three plots. Wheat leaf samples were collected in triplicate from both rehydrated and continuous-drought-stressed seedlings at 1 (169 DAS), 8 (176 DAS), 15 (183 DAS) days after irrigation. Leaf samples collected 1 day before the rehydration served as control [167 DAS, 0 days post-rehydration (dpr)]. The soil water content of 0–60 cm layer in the rehydrated field was greater than 25% at 15 dpr, indicating a sufficient water supply for wheat growth. Soil water content of 0–60 cm layer in the continuous-drought-stressed field was consistently lower than 20% (Figure 1C).

Plant height was determined by measuring the main tiller at 15 dpr (183 days after sowing). To monitor the development of the wheat embryos, developing spikes were collected at 1, 8, and 15 dpr with awns and rachis removed. The isolated embryos were observed using anatomic microscope. For evaluation of physiological activities, quantification of phytohormone content, RNA sequencing, and qRT-PCR assay, samples were collected from the fully expanded fourth leaves of wheat plants at 1, 8, and 15 dpr (Figure 1B). Leaf samples from 10 independent plants were combined as one biological replicate. Three biological replicates were collected at each time point. All the samples were immediately frozen in liquid nitrogen and stored at −80°C.

### Physiological Traits Measurement

The SOD activity was measured using the nitro-blue tetrazolium photoreduction method (Giannopolitis and Ries, 1977). The POD activity was measured using the guaiacol method (Han et al., 2008). The CAT activity was estimated by measuring the initial disappearance rate of H_2_O_2_ (Shimizu, 1987). The APX activity was measured using the method from Asada (1980). The MDA content was determined using the thiobarbituric acid reaction as described previously (Bramlage, 1992). The soluble sugars content was evaluated using the anthrone colorimetric method (Miller, 1959). The proline content was quantified using the ninhydrin colorimetric method (Bates et al., 1973). The soluble protein content was measured using the Coomassie brilliant blue method (Campion et al., 2011).

### Phytohormone Content Quantification

Five endogenous phytohormones in wheat leaves, including ABA, IAA, GA, SA, and JA, were quantified using the high-performance liquid chromatography (HPLC) method. Briefly, 2 g fresh wheat leaves were weighed and ground into a slurry with 10 ml pre-cooled 80% methanol in an ice bath. This slurry was sealed in plastic wrap and kept overnight at 4°C. The supernatant was obtained by centrifugation at 8,000 g at 4°C for 10 min. The residue was re-suspended in 8 ml pre-cooled 80% methanol, and the supernatant was collected again by centrifugation. The filtrate was concentrated under reduced pressure at 40°C to one-third of the original volume. Samples were extracted and decolorized using 30 ml petroleum ether three times, with the ether phase discarded each time. The aqueous phase was extracted using 20 ml ethyl acetate three times, and the ester phases were combined. Samples were then dried by evaporation under reduced pressure at 40°C. Samples were mixed with 2 ml acetic acid solution (pH 3.5), purified using a Sep-Pak C18 cartridge, eluted with methanol, and concentrated to dry powders under reduced pressure at 40°C. Before high-performance liquid chromatography (HPLC), samples were dissolved, diluted to a total volume of 2 ml in a mobile phase, and filtered through a 0.45 μm microporous membrane. An Agilent 1260 High Performance Liquid Chromatograph was employed. The chromatographic column used was an Eclipse XDB-C18 (250 mm × 4.6 mm, 5 μm, Agilent, CA, United States). Mobile phase A was methanol, and phase B was an aqueous acetic acid solution (pH 3.5). Five endogenous phytohormones in wheat leaves were separated at the same time by gradient elution. The gradient conditions were set as 0–7 min, 20% A; 7–10 min, 20–28% A; 0–17 min, 28% A; 17–19 min, 28–40% A; and 19–35 min, 40% A. The flow rate was 1 mL/min, and the injection volume was 10 μL. The detector wavelengths were set to 254 and 240 nm, and the detection temperature was room temperature (Zhong et al., 2013).

### RNA-Seq and Bioinformatics Analysis

Total RNA was extracted using TRIzol reagent (Invitrogen, Thermo Fisher Scientific, United States) in accordance with the manufacturer’s instructions. Sequencing libraries were generated using NEBNext ^®^ Ultra™ RNA Library Prep Kit for Illumina ^®^ (NEB, MA, United Kingdom) following the manufacturer’s recommendations. First-strand cDNA was synthesized using a random hexamer primer and M-MuLV Reverse Transcriptase (RNase H-). Second-strand cDNA synthesis was subsequently performed using DNA Polymerase I and RNase H. PCR products were purified (AMPure XP system), and library quality was assessed on the Agilent Bioanalyzer 2100 system. Q20, Q30, and the GC content of the clean data were calculated. Clean reads were aligned to wheat reference genome sequences released by the International Wheat Genome Sequencing Consortium using HISAT2 (Kim et al., 2015).

To assess the variability among samples, we performed a principal component analysis for the overall transcripts of each sample using the prcomp command line with default parameters in the R software package (Robinson et al., 2010). The comparisons were made between rehydration and control samples at 1, 8, and 15 dpr. An adjusted *p*-value (*q*-value) < 0.05 and |log2FoldChange| ≥ 1 were set as the criteria for determining DEGs and classified into clusters using k-means clustering. Heatmaps were visualized using the R package Complex Heatmap v2.5.5 (Yi et al., 2019). Gene ontology (GO) and Kyoto Encyclopedia of Genes, and Genomes (KEGG) pathway enrichments of DEGs were conducted using the R software package based on the hypergeometric distribution. Raw data for the transcriptome were deposited at NCBI BioProject PRJNA774165.

### qRT-PCR

Total RNA was extracted from samples using a QIAGEN Plant RNA extraction kit (QIAGEN, Hilden, Germany). The first-strand cDNA was synthesized using the EasyScript First-Strand cDNA reagent (Transgen Biotech, Dalian, China). The wheat *Actin* gene (GenBank accession AB181991, primers in Supplementary Table 1) was used as an internal reference (Rong et al., 2016). Six genes (wheat genome accessions *TraesCS7D02G199900*, *TraesCS7A02G337000*, *TraesCS6A02G094400*, *TraesCS3A02G535300*, *TraesCS3A02 G081300*, and *TraesCS2D02G340100*) were randomly selected from DEGs for the qRT-PCR assay to evaluate the reliability of the expression levels determined by RNA-seq. Primers for these DEGs were designed (Supplementary Table 1). Three biological replicates were included for each treatment. qRT-PCR was performed using the PerfectStart™ Green qPCR SuperMix (Transen Biotech, Beijing, China). The threshold values (Ct) were calculated by the Roche LightCycler 96 (Roche, Indianapolis, IN, United States). Gene expression was evaluated using the 2^–ΔΔCt^ method (Livak and Schmittgen, 2001). The FPKM values of these six DEGs were collected from the transcriptome database to profile the expression patterns. The correlation of overall expression levels of these six genes between qRT-PCR and RNA-seq was calculated using the R software package.

### Data Processing and Statistical Analysis

All of the statistical analyses were performed using SPSS Statistics 22.0 (IBM, NY, United States). Data are presented as means ± standard error (SE) values. A one-way ANOVA was conducted, and Student’s *t*-tests were used to compare treatment means at the 5% level.

## Results

### A Drought-Tolerant Winter Wheat Cultivar Showed Better Rehydration-Compensation Phenotypes Than a Drought-Susceptible Cultivar

**FIGURE 2:**
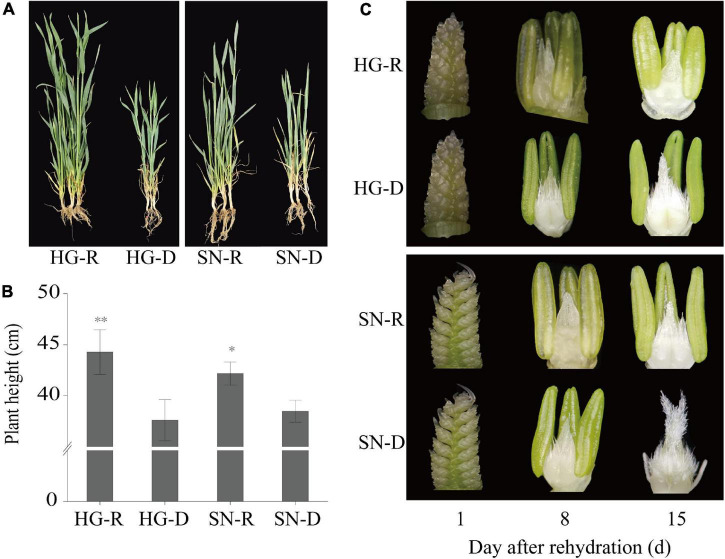
The drought-tolerant wheat variety “Hengguan35” (HG) showed better rehydration-compensation phenotypes than the drought-sensitive wheat variety “Shinong086” (SN). **(A)** Plant morphology of HG and SN at 15 dpr. “*, **” indicate significant difference at 0.05 level. **P < 0.01; *P < 0.05. **(B)** Plant height was measured at 15 dpr. **(C)** Spikes were isolated and observed using a microscope at 1, 8, and 15 dpr. R, rehydrated plants; D, plants under continuous drought stress.

To investigate whether there is an association between rehydration compensation and drought tolerance, we compared the growth of drought-tolerant cultivar “HG” and the drought-sensitive cultivar “SN” under rehydration and drought-stress conditions (Figure 1). Plants from both watered (rehydrated, R) and non-watered (continuous drought stress, D) field were collected and photographed at 15 dpr (Figure 2A). The growth of drought-stressed plants of both HG and SN were recovered upon rehydration. Plant heights significantly increased after the rehydration treatment, and the drought-tolerant cultivar showed a pronounced faster growth than the drought-sensitive cultivar (Figure 2B). We further checked embryonic development of these wheat plants at 1, 8, 15 dpr (Figure 2C). The ear development was postponed by the rehydration treatment, with much greater delays in HG than SN. Thus, the drought-tolerant winter wheat cultivar appeared to possess a better rehydration compensation by promoting plant growth and postponing spike development.

### Rehydration Alleviated Peroxidation and Osmotic Stress

**FIGURE 3:**
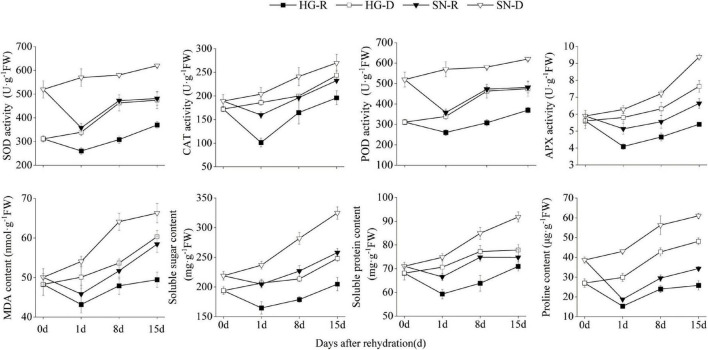
Physiological activities in HG and SN upon rehydration and continuous drought stress. The activity levels of SOD, POD, CAT, and APX in both HG and SN at 0, 1, 8, and 15 dpr were measured. The dynamic changes in contents of MDA, Pro, soluble protein, and soluble sugars in these samples were also monitored.

To characterize the physiological changes of wheat leaves after being rehydrated, we further measured the SOD, POD, catalase (CAT), and ascorbate peroxidase (APX) activity levels in both HG and SN at 0, 1, 8, and 15 dpr (Figure 3). The overall SOD, POD, CAT, and APX activities were suppressed after rehydration, and were much lower in the HG than SN. We also measured the dynamic changes in MDA, Pro, soluble protein, and soluble sugars levels (Figure 3). Interestingly, all these stress-related contents were reduced after rehydration, and the overall levels of them were lower in HG than those in SN. These results indicated that rehydration process alleviated peroxidative activities and reduced stress-related contents.

### Phytohormone Contents of Abscisic Acid, Gibberellin, Jasmonic Acid, and Salicylic Acid Were Reduced Upon Rehydration

**FIGURE 4:**
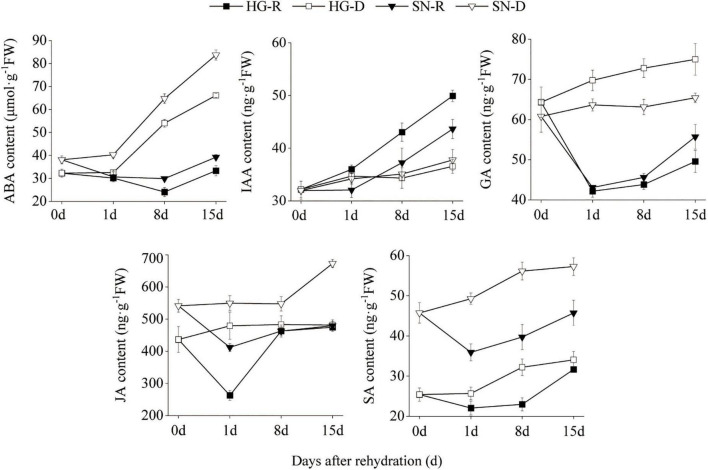
The contents of phytohormones in HG and SN upon rehydration and continuous drought stress were evaluated using HPLC. The concentrations of ABA, IAA, GA, JA, and SA in both HG and SN leaves at 0, 1, 8, and 15 dpr were quantified.

To determine the key phytohormones involved in the rehydration process, we quantified the concentrations of ABA, IAA, GA, JA, and SA using HPLC in both HG and SN leaves at 0, 1, 8, and 15 dpr (Figure 4). We observed that the ABA content increased gradually under continuous drought stress, whereas decreased in both SN and HG upon rehydration at 1 and 8 dpr. Moreover, the overall ABA content in HG was significantly lower than SN in either rehydration or drought treatment. The IAA content was accumulated during continuous drought stress, with even higher level in HG than SN. The GA content was reduced in both HG and SN upon rehydration. In the continuous drought treatment, the overall GA content in HG was significantly higher than SN. JA content in HG was significantly lower than SN before the rehydration treatment (0 dpr). The JA content was greatly reduced upon rehydration treatment in both HG and SN at 1 dpr, and then increased rapidly at 8 dpr. The overall SA content in HG was significantly lower than SN in either rehydration or drought treatment. Upon rehydration treatment, the SA content was decreased in both HG and SN at 1 dpr, and then increased gradually at 8 and 15 dpr. These results indicated that phytohormone accumulations in HG and SN differed before the rehydration treatment, which may result from the drought-tolerant features of these cultivars. The rehydration process was associated with the decreased accumulations of ABA, GA, JA, and SA.

### Transcriptome Sequencing Revealed the Molecular Mechanisms of Rehydration

**FIGURE 5:**
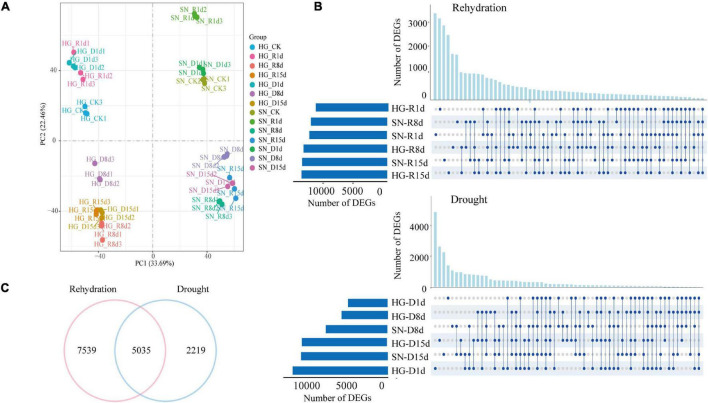
Transcriptome responses of winter wheat upon rehydration and continuous drought stress. **(A)** A principal component analysis for the overall gene expression levels in all the sequenced RNA libraries was conducted to evaluate the correlations among different samples. **(B)** Upset diagrams showing the DEGs at 1, 8, and 15 dpr. The lines between the two points represent the specific expressed genes between the samples, and the length of the columns represent the number of genes. **(C)** Venn diagram showing numbers of rehydration-drought shared, rehydration-specific, and drought-specific DEGs.

RNA samples were collected from leaves of HG and SN at 0, 1, 8, and 15 dpr. Each sample included three biological replicates. In general, 42 RNA samples were prepared and sent for 12-Gb Illumina sequencing (Supplementary Table 2). The *Triticum aestivum* reference genome (IWGSCV1.1 version) from Ensembl Genomes was used to assemble the transcriptome (Supplementary Table 3). In total, 132,982 genes were mapped to the genome sequence. A principal component analysis revealed a high degree (*R*^2^ > 0.92) of correlation in the overall gene expression levels among biological replicates (Supplementary Figure 1). Samples were clustered based on genotype (HG or SN) and time point (0, 1, 8, or 15 dpr), respectively (Figure 5A). The fragments per kilobase of transcript per million mapped reads (FPKM) value was used to estimate the expression levels of the genes in the transcriptome. The differentially expressed genes (DEGs, *q*-value < 0.05, |log2foldchange| > 1) were identified using DESeq2. All the raw data for the transcriptome assembly have been deposited in NCBI under BioProject PRJNA774165.

Compared with the control group before rehydration (0 dpr), we identified 12,073, 13,927, and 14,250 DEGs in rehydrated HG at 1, 8, and 15 dpr, respectively. A total of 12,908, 12,079, and 14,169 DEGs were found in rehydrated SN at 1, 8, and 15 dpr, respectively. Compared with samples from HG at 0 dpr, there were 12,592, 6,117 and 11,039 DEGs in the non-watered HG (continuous drought stress) at 1, 8, and 15 dpr, respectively. There were 5,224, 8,151 and 11,478 DEGs in the non-watered SN at 1, 8, and 15 dpr, respectively. Interestingly, a large number of these identified DEGs (approximately 40%) encoded transcription factors (TFs), indicating a dynamic transcriptional change was triggered by the rehydration treatment (Supplementary Table 4).

### Rehydration Activated Biosynthetic Processes and Suppressed Stress-Response Pathway

To further explore the core DEGs associated with rehydration or continuous drought stress, we checked the co-regulated DEGs between HG and SN at each time point. We identified 3,969, 6,964, and 6,230 co-regulated DEGs in both rehydrated HG and SN at 1, 8, and 15 dpr, respectively (Supplementary Figure 2). We combined all these genes and identified 12,574 non-redundant rehydration responsive DEGs. On the other hand, a total of 2,070, 2,492, and 5,087 co-regulated DEGs in both non-watered HG and SN were identified at 1, 8, and 15 dpr, respectively (Figure 5B and Supplementary Figure 2). Combined from these genes, a total of 7,254 non-redundant drought responsive DEGs were identified. Interestingly, our further analysis revealed 5,035 shared genes between rehydration responsive DEGs and drought responsive DEGs (Figure 5C). Meanwhile, a total of 7,539 genes were designated as rehydration-specific DEGs and 2,219 genes as drought-specific DEGs.

**FIGURE 6:**
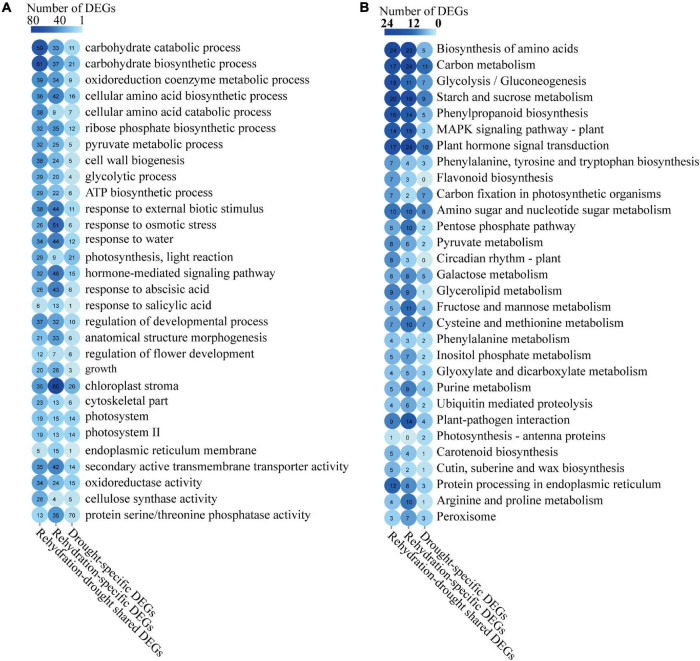
GO and KEGG annotations of the rehydration-drought shared, rehydration-specific, and drought-specific DEGs. Enrichment of GO annotations **(A)** and KEGG pathways **(B)** in rehydration-drought shared, rehydration-specific, and drought-specific DEGs were summarized.

A GO enrichment analysis was conducted on these rehydration-drought shared, rehydration-specific and drought-specific DEGs (Figure 6A). Rehydration-drought shared DEGs were enriched in carbohydrate biosynthetic process, carbohydrate catabolic process, and oxidoreduction coenzyme metabolic process. Rehydration-specific DEGs were enriched in chloroplast stroma, response to osmotic stress, and hormone-mediated signaling pathway. Drought-specific DEGs were involved in chloroplast stroma, carbohydrate biosynthetic process, and photosynthesis. More genes with GO annotations related to hormone-mediated signaling pathways, including genes response to ABA and SA, were annotated in rehydration-specific DEGs than drought-specific DEGs.

KEGG annotations of these rehydration-drought shared, rehydration-specific and drought-specific DEGs were summarized to illustrate the regulatory pathways (Figure 6B). We found DEGs in all these three analysis groups were enriched in KEGG pathways of carbon metabolism, plant hormone signal transduction, and starch and sucrose metabolism. Compared with drought-specific DEGs, more genes in KEGG pathways of biosynthesis of amino acids, phenylpropanoid biosynthesis, and mitogen-activated protein kinase (MAPK) signaling pathway were annotated in rehydration-drought shared and rehydration-specific DEGs.

### Suppression of Genes Associated With Abscisic Acid Signaling Transduction Were Much Stronger in Hengguan35 Than in Shinong086 Upon Rehydration Treatment

**FIGURE 7:**
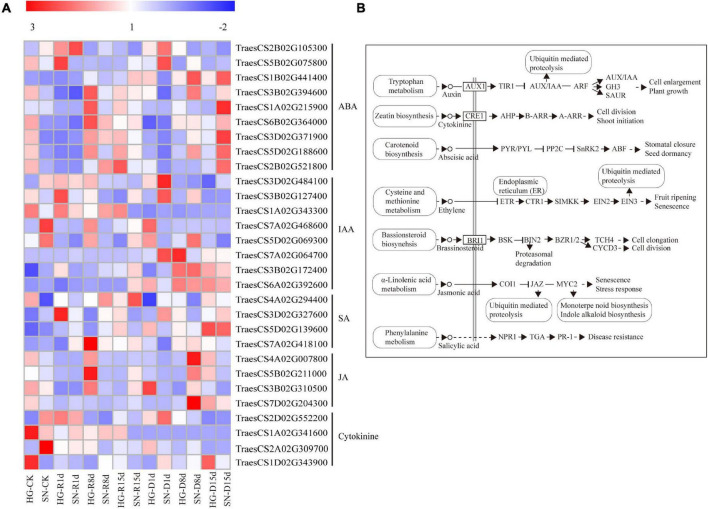
Expression profiles of DEGs involved in plant hormone pathways. The expression profiles of transcripts **(A)** involved in hormone signal transduction pathways **(B)** were generated using their FPKM values.

To reveal the molecular mechanisms controlling rehydration compensation in HG, we analyzed the expression levels of genes enriched in the “plant hormone signal transduction” KEGG pathway (Figure 7). Two *Triticum aestivum* sucrose non-fermenting-1-related protein kinase 2 genes (*TaSnRK2*, *TraesCS1A02G215900* and *TraesCS2B02G521800*) were significantly suppressed in HG at all three rehydration time points. A pyraclostrobin (*PYR*) or pyrabactin-resistance1-like (*PYL*) transcription factor gene (*TraesCS2B02G105300*) was upregulated at 8 dpr in both HG and SN, with a longer duration of induction in HG at 15 dpr. Three protein phosphatase type 2C genes (*PP2C*, *TraesCS1B02G441400*, *TraesCS1B02G441400*, and *TraesCS5D02G188600*) and two ABA-responsive element-binding factor genes (*TraesCS6B02G364000* and *TraesCS3D02G371900*) were downregulated upon rehydration, with a more dramatic decrease in HG than SN. Moreover, the expression levels of several genes involved in the IAA, JA, and SA signaling pathways were also significantly altered.

### Hengguan35 Kept a More Balanced Expression of Genes Involved in ROS Pathway Than Shinong086

**FIGURE 8:**
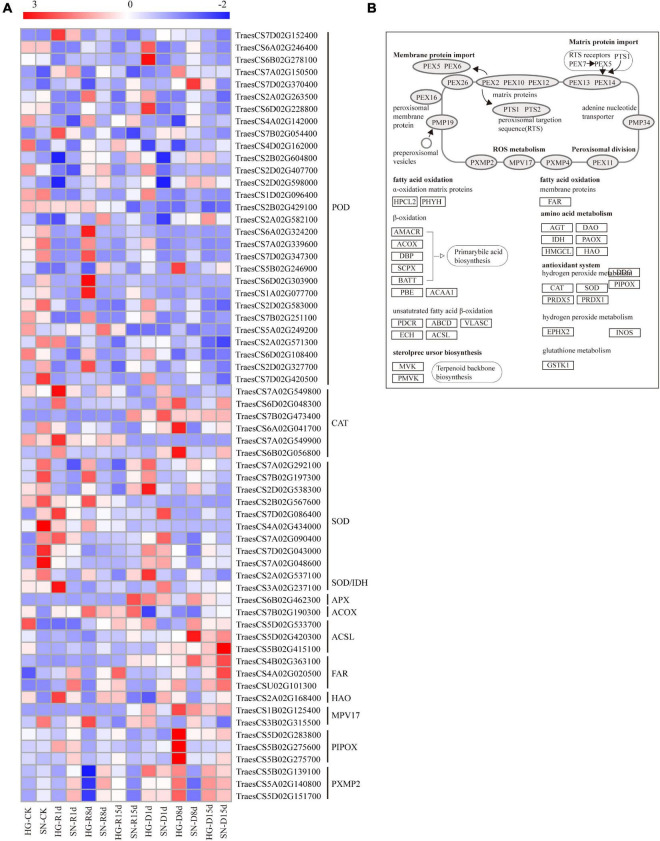
Expression profiles of DEGs involved in ROS-related pathways. The expression levels of transcripts **(A)** in antioxidative and biosynthetic pathways **(B)** were generated using their FPKM values.

We further analyzed DEGs involved in the ROS network to interpret their associations with oxidation reduction and acclimation during rehydration compensation (Figure 8). The overall expression levels of three peroxisomal membrane protein 2 genes (*PXMP2*, *TraesCS5B02G139100*, *TraesCS5A02G140800*, and *TraesCS5D02G151700*) were downregulated upon rehydration, and had lower expression levels in HG compared with SN. The mean platelet volume gene (*Mpv*, *TraesCS1B02G125400*) controlling ROS metabolism had accumulated transcripts in SN but was suppressed in HG. One of the Long-chain acyl-CoA synthetases genes (*ACSL*, *TraesCS5D02G533700*) was constantly downregulated in HG after the rehydration treatment, whereas it was only downregulated at 8 dpr in SN. Three SOD related genes (*TraesCS4A02G434000*, *TraesCS7D02G043000*, and *TraesCS7A02G048600*) were significantly upregulated at the early stage of rehydration. Most of the CAT related genes were induced at the late stage of rehydration. Interestingly, the overall expression level of one CAT related gene, *TraesCS7B02G473400*, in HG was significantly lower than that in SN, whereas the other CAT related gene, *TraesCS7A02G549900*, showed an opposite expression pattern. Based on the expression profiles of these genes involved in the ROS network, we speculated that HG might utilize a more balanced ROS pathway to achieve a greater rehydration compensation level.

### Validation of Gene Expression by qRT-PCR

**FIGURE 9:**
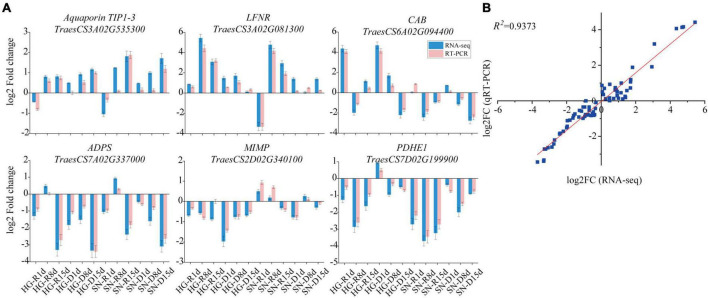
The expression profiles of six randomly selected DEGs were validated by qRT-PCR assay. **(A)** The expression patterns of six randomly selected DEGs were generated using both qRT-PCR data and FPKM values. The relative transcript abundances were expressed relative to that of the internal reference *TaActin* following the 2^–ΔΔCt^ method. FPKM values of these DEGs were collected from the transcriptome database. **(B)** Correlation analysis on the overall expression levels of six selected DEGs between RNA-seq and qRT-PCR. Log2FC values of RNA-seq data (x-axis) were plotted against log2FC values of qRT-PCR data (y-axis). R, rehydrated plants; D, plants under continuous drought stress.

To validate the expression patterns of genes determined by RNA-seq, Aquaporin TIP1-3 (*TraesCS3A02G535300*), leaf type ferredoxin-NADP and oxidoreductase (LFNR, *TraesCS3A02G081300*), Chlorophyll *a/b* binding protein (*CAB*, TraesCS6*A02G094400*), alpha-D-phosphohexomutase superfamily (*ADPS*, *TraesCS7A02G337000*), Mitochondrial inner membrane protease ATP23 (*MIMP*, *TraesCS2D02G340100*), and Pyruvate dehydrogenase E1 component subunit alpha (*PDHE1*, *TraesCS7D02G199900*) were randomly selected from DEGs for a quantitative real-time PCR (qRT-PCR) analysis (Figure 9A). The wheat *TaActin* gene (GenBank accession AB181991.1) was used as an internal control. A high correlation (*R*^2^>0.9373) between overall expression levels from RNA-seq and qRT-PCR assays was observed (Figure 9B).

## Discussion

Winter wheat is susceptible to water scarcity, and high yields are normally associated with a sufficient water supply (Johnson et al., 2018). At present, wheat farmers have to consider both plant productivity and the costs of changing environmental factors (Xu et al., 2018; Yu et al., 2020). Monitoring plant morphological and biochemical changes upon rehydration after drought stress is important for characterizing drought-tolerant wheat genotypes (Jiang et al., 2020). In response to drought stress, plants adjust their flowering times to adapt to the vegetative growth stage (Shavrukov et al., 2017). Growth inhibition is an early response to drought stress (Kumar Jha et al., 2019). Rehydration at different growth stages can compensate for the decline in biomass caused by crop water deficit, especially after a moderate drought before the booting stage (Jiang et al., 2020). There is an obvious compensatory phenomenon in wheat plant height and leaf area (Li Y. et al., 2020), but there are rare reports of ear development on winter wheat after rehydration. In this work, we monitored the compensatory growth of two winter wheat varieties that have different drought resistance levels after rehydration (Figure 1). The promotion of plant growth and postponement of spike development were observed in winter wheat cultivars after rehydration. Moreover, the drought-tolerant cultivar showed a greater rehydration compensation than the drought-sensitive cultivar (Figure 2). For similar experiments in other crops, the elongation of young leaves in maize is extremely sensitive to the water supply and a rehydration treatment in the root medium immediately accelerates the growth of young leaves (Jiang et al., 2020). Rehydration compensation also delays the growth period to relieve the negative impacts of drought stress. It is considered a self-defense response of plants against short-term, periodic, or unpredictable drought (Mu et al., 2021). In addition, the compensation effect is largely dependent on the strength and duration of the stress (Rajala et al., 2009; Guo et al., 2014). For example, compared with the normally watered control, plant height increases and leaf area expands dramatically in soybean after rehydration (Dong et al., 2019).

Rehydration after drought stress induces complex biological processes in plant that require an energy balance between growth and stress avoidance (Zhu, 2016; Gupta et al., 2020). In plants, drought stress induces a wide range of responses, including increasing the oxidative damage in chloroplasts, inhibiting photosynthesis, suppressing metabolic responses, activating glycocatabolism, and changing cellular lipid compositions (Mittler and Blumwald, 2015; Yi et al., 2016; Li et al., 2019; Hrmova and Hussain, 2021; Waititu et al., 2021). ABA is the key phytohormone that mediates drought tolerance and rehydration compensation in plants (Saradadevi et al., 2017; Mega et al., 2019; Zhang et al., 2021). Upon rehydration, the levels of ABA and several other hormones, including IAA, GA, and cytokinin, first decline rapidly and then rise slowly, resulting in a “V”-shaped changing pattern (Dong et al., 2019). In the current investigation, we found dramatic decreases in the contents of ABA, GA, JA, and SA upon rehydration (Figure 4). Genes encoding ABA receptors, including *PYR*, *PYL*, receptor component of ABA receptor (*RCAR*) and *PP2C*, were differentially expressed after rehydration treatment as evidenced in the RNA-seq database (Figure 7). SnRK2 is a key kinase that phosphorylates many ABA downstream proteins (Lin et al., 2021). PP2C keeps SnRK2 inactive by blocking its catalytic cleft and de-phosphorylating its activation loop. However, the phosphorylation activity of PP2C is inhibited by ABA-PYL (Mosfeq-Ul Hasan et al., 2019; Li et al., 2021). Interactions between PP2C and ABA-PYL eventually release SnRK2. In our transcriptome database, the *TaPYR/PYL* gene was upregulated, whereas both the *TaPP2C* and *TaSnRK2* genes were downregulated after the rehydration treatment, indicating a suppression of the ABA pathway during rehydration compensation. Moreover, we noticed that expression levels of several ABA-signaling genes were much lower in HG than in SN. Combined with evidence showing that the ABA content in HG was significantly lower than in SN, we speculated that drought-tolerant wheat maintained a lower ABA content during drought stress and suppressed ABA signaling transduction upon rehydration.

Almost all abiotic stresses lead to the regulation of secondary oxidative stress stimulatory signals in a tissue-specific or even cell-specific manner (Chaves and Oliveira, 2004). Increases in the corresponding enzyme activities under stress conditions protects plant cells from oxidative damage (Chen et al., 2017; Zhang L. et al., 2020). To protect enzyme systems and membranes from lipid peroxidation, SOD, POD, and MDA activities increased at each growth stage after drought stress (Gill and Tuteja, 2010; Abid et al., 2018). In this study, contents of the soluble protein, soluble sugar, and Pro, as well as the activities of SOD, CAT, and POD, increased under drought stress, decreased rapidly upon rehydration and then increased gradually with the extension of rehydration (Figure 3). These dynamic changes were consistent with the results of previous investigations on corn, cotton, and other crops (Furlan et al., 2016; Voronin et al., 2019; Xu et al., 2019). Fluctuations in ROS are sensed by receptor proteins NADPH cytochrome P450 reductase (NPR) and heat stress response (*HSF*), redox-sensitive transcription factors, or phosphatases. Serine/threonine-protein kinases are involved in ROS induction, including the activation of mitogen-activated protein kinase, which further regulates the ROS-signaling network (Pitzschke and Hirt, 2009; Liu and He, 2017). The expression levels of genes in the ROS pathway are quantitatively influenced by the accumulation of ROS. In cotton, some transcription factor families, such as *MYB*, *WRKY*, *AP2/ERF*, and *NAC*, are involved in regulating plant responses to abiotic stress and ROS-signaling network (Mosfeq-Ul Hasan et al., 2019). In rice, myo-inositol oxygenase (*MIOX*) family genes significantly improved the scavenging activity of ROS under drought stress (Duan et al., 2012). In this study, we observed association between the expression changes of ROS-related genes and rehydration treatment. Moreover, HG kept a more balanced expression of ROS-related genes than SN (Figure 8). Therefore, we speculate that rehydration of winter wheat induces the expression of ROS-related genes, reduces the contents of SOD, CAT, and MDA, and eventually diminishes the oxidative damage caused by drought stress.

## Conclusion

In conclusion, we initially profiled the dynamic changes in physiological responses and gene expressions associated with rehydration in winter wheat under field conditions. Rehydration significantly promoted winter wheat growth and postponed spike development. A drought-tolerant variety showed a greater growth compensation than a drought-sensitive variety, which may be resulted from lower level of stress-related phytohormones and activity of oxidoreductase. Genes involved in ABA signaling pathway and ROS balancing are playing crucial roles in the rehydration process. Our study provides new insights into the mechanism of rehydration compensation in crops and valuable gene resources for the genetic improvement of wheat.

## Data Availability Statement

The original contributions presented in the study are publicly available. This data can be found here: National Center for Biotechnology Information (NCBI) BioProject database under accession number PRJNA774165.

## Author Contributions

WZ conceived the project and set the scientific objectives. XL, XW, PL, XB, and XH contributed to the preparation of equipment and acquisition of data. XL, MY, and XW wrote the manuscript. All authors have read and approved the published version of the manuscript.

## Conflict of Interest

The authors declare that the research was conducted in the absence of any commercial or financial relationships that could be construed as a potential conflict of interest.

## Publisher’s Note

All claims expressed in this article are solely those of the authors and do not necessarily represent those of their affiliated organizations, or those of the publisher, the editors and the reviewers. Any product that may be evaluated in this article, or claim that may be made by its manufacturer, is not guaranteed or endorsed by the publisher.
