# Supplementary material for: Rehydration Compensation of Winter Wheat Is Mediated by Hormone Metabolism and De-Peroxidative Activities Under Field Conditions
Source: Front Plant Sci. 2022 Feb 24;13:823846. doi: 10.3389/fpls.2022.823846 (PMC8908233; doi:10.3389/fpls.2022.823846)
Supplement: Supplementary file 1 [file Data_Sheet_1.docx]

Supplementary Material

## Supplementary Figures


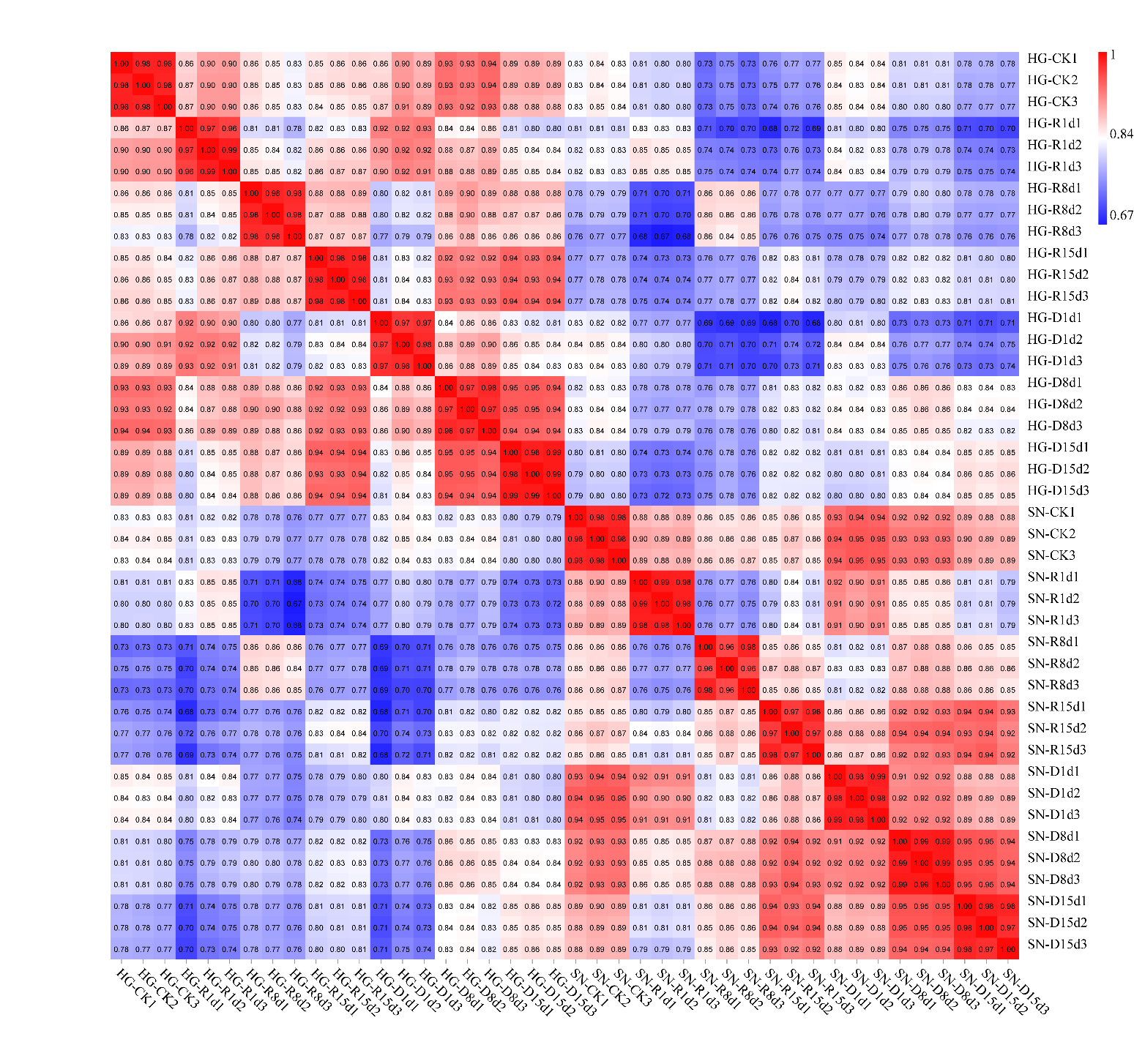


**Figure S1.** Pearson’s correlations of the overall gene expression levels among different samples.
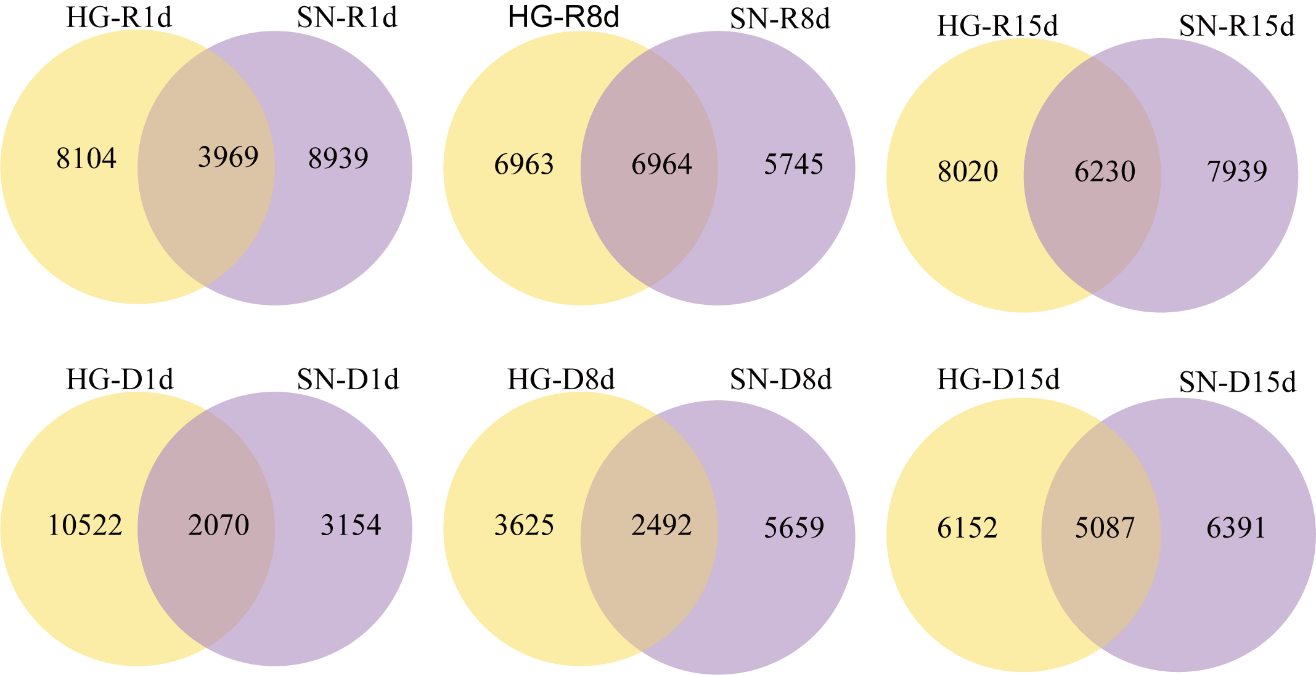


**Figure S2.** Venn diagrams of DEGs in HG and SN at 1, 8, and 15 dpr.

## Supplementary Tables

**Table S1.** The qRT-PCR primers of six randomly selected DEGs.

| **ID** | **Primer_F** | **Primer_R** |
| --- | --- | --- |
| *TraesCS2D02G340100* | AACGGGAACTGATGAAAGGCT | GACAGATTCCAGCGAACGTC |
| *TraesCS3A02G081300* | TACAGCTCCAGCGTCTCGTA | CTTCGTCTCCGGGTACATCTG |
| *TraesCS3A02G535300* | TGTGTGCGAGTCAGTCAGTC | GGCAAGACATCAAGTTGCTAGG |
| *TraesCS6A02G094400* | GACCCCGTCAACAACAATGC | AGTGGACAACTCAAGCCGTG |
| *TraesCS7A02G337000* | TGAGTTCGTTGACACCAGCG | AGAACCAAAAGGCATTCCCCA |
| *TraesCS7D02G199900* | TGCCCGGATTGAAGGTTGAT | AGTACGGTAAGTGCTGCCTG |
| *Actin* | ACCTTCAGTTGCCCAGCAAT | CAGAGTCGAGCACAATACCAGTTG |

**Table S2.** Sequencing information of the RNA-seq samples.

| **Sample name** | **Raw reads** | **Clean reads** | **Clean bases** | **Error rate(%)** | **Q20(%)** | **Q30(%)** | **GC content(%)** |
| --- | --- | --- | --- | --- | --- | --- | --- |
| HG-CK1 | 93255696 | 91980754 | 13.80G | 0.03 | 97.54 | 93.28 | 56.00 |
| HG-CK 2 | 112099494 | 110912648 | 16.64G | 0.03 | 97.6 | 93.26 | 55.89 |
| HG-CK 3 | 119798524 | 118415530 | 17.76G | 0.03 | 97.48 | 93.15 | 56.44 |
| HG-R1d1 | 121470180 | 119469232 | 17.92G | 0.03 | 97.85 | 93.90 | 56.91 |
| HG-R1d 2 | 117187232 | 115639888 | 17.35G | 0.03 | 97.50 | 93.16 | 56.18 |
| HG-R1d 3 | 94015742 | 92862576 | 13.93G | 0.03 | 97.25 | 92.64 | 56.40 |
| HG-R8d1 | 108203318 | 107205762 | 16.08G | 0.03 | 97.67 | 93.45 | 56.46 |
| HG-R8d2 | 104466474 | 103232946 | 15.48G | 0.03 | 97.75 | 93.63 | 57.45 |
| HG-R8d3 | 88746666 | 87818198 | 13.17G | 0.03 | 97.68 | 93.6 | 56.92 |
| HG-R16d1 | 102085458 | 100957668 | 15.14G | 0.03 | 97.66 | 93.47 | 54.58 |
| HG-R16d2 | 107087344 | 106049098 | 15.91G | 0.03 | 97.64 | 93.69 | 55.34 |
| HG-R16d3 | 145081640 | 143643334 | 21.55G | 0.03 | 97.56 | 93.27 | 54.69 |
| HG-D1d1 | 97888080 | 97088070 | 14.56G | 0.03 | 97.55 | 93.21 | 56.93 |
| HG-D1d2 | 90272454 | 89492142 | 13.42G | 0.03 | 97.63 | 93.34 | 57.08 |
| HG-D1d3 | 126846550 | 125149694 | 18.77G | 0.02 | 98.04 | 94.34 | 58.02 |
| HG-D8d1 | 132855304 | 131607848 | 19.74G | 0.03 | 97.38 | 92.87 | 56.28 |
| HG-D8d2 | 114714456 | 113351812 | 17.00G | 0.03 | 97.64 | 93.69 | 57.58 |
| HG-D8d3 | 120229688 | 117955892 | 17.69G | 0.03 | 97.63 | 93.46 | 56.74 |
| HG-D16d1 | 139525392 | 137432406 | 20.61G | 0.03 | 97.48 | 93.12 | 54.5 |
| HG-D16d2 | 136078662 | 134214208 | 20.13G | 0.03 | 97.6 | 93.29 | 54.68 |
| HG-D16d3 | 158975270 | 156836398 | 23.53G | 0.03 | 97.43 | 92.98 | 53.99 |
| SN-CK1 | 105320060 | 103580818 | 15.54G | 0.03 | 97.52 | 93.2 | 56.42 |
| SN-CK 2 | 101006106 | 99416036 | 14.91G | 0.03 | 97.57 | 93.29 | 56.71 |
| SN-CK 3 | 110888182 | 109203736 | 16.38G | 0.03 | 97.98 | 94.13 | 56.34 |
| SN-R1d1 | 110641040 | 109280530 | 16.39G | 0.03 | 97.17 | 92.52 | 56.41 |
| SN-R1d 2 | 85555192 | 84413250 | 12.66G | 0.03 | 97.53 | 93.24 | 56.07 |
| SN-R1d 3 | 94181880 | 93013924 | 13.95G | 0.03 | 97.49 | 93.20 | 55.60 |
| SN-R8d1 | 119413120 | 117563262 | 17.63G | 0.03 | 97.86 | 93.86 | 56.78 |
| SN-R8d2 | 99069248 | 97765352 | 14.66G | 0.03 | 97.53 | 93.21 | 56.35 |
| SN-R8d3 | 106246312 | 105158986 | 15.77G | 0.03 | 97.52 | 93.17 | 56.22 |
| SN-R16d1 | 103452014 | 102378656 | 15.36G | 0.03 | 97.61 | 93.33 | 56.01 |
| SN-R16d2 | 106437050 | 105358876 | 15.80G | 0.03 | 97.81 | 94.01 | 56.78 |
| SN-R16d3 | 115338662 | 113556996 | 17.03G | 0.03 | 97.57 | 93.28 | 56.00 |
| SN-D1d1 | 96065096 | 94626948 | 14.19G | 0.03 | 97.70 | 93.57 | 56.85 |
| SN-D1d2 | 109717844 | 108098612 | 16.21G | 0.03 | 97.63 | 93.38 | 56.73 |
| SN-D1d3 | 114784076 | 113288258 | 16.99G | 0.03 | 97.56 | 93.22 | 56.02 |
| SN-D8d1 | 107021448 | 105621880 | 15.84G | 0.03 | 97.46 | 92.99 | 55.79 |
| SN-D8d2 | 113915656 | 112517068 | 16.88G | 0.03 | 97.40 | 92.85 | 56.42 |
| SN-D8d3 | 107434108 | 105902248 | 15.89G | 0.03 | 97.43 | 93.03 | 56.16 |
| SN-D16d1 | 117678084 | 116332586 | 17.45G | 0.03 | 97.47 | 93.05 | 55.28 |
| SN-D16d2 | 128491624 | 126656332 | 19.00G | 0.03 | 97.33 | 92.77 | 55.74 |
| SN-D16d3 | 123843806 | 122221430 | 18.33G | 0.03 | 97.54 | 93.22 | 55.54 |

HG, drought-tolerant wheat variety ‘Hengguan35’; SN, drought-sensitive wheat variety ‘Shinong086’. R, rehydrated plants; D, plants under continuous drought stress.

**Table S3.** Transcriptome assembly information of the RNA-seq samples.

| **Sample name** | **Total reads** | **Total mapped**  **(%)** | **Multiple mapped (%)** | **Uniquely mapped (%)** | **Read-1**  **(%)** | **Read-2**  **(%)** | **Reads map to '+'(%)** | **Reads map to '-'(%)** | **Non-splice reads(%)** | **Splice reads(%)** |
| --- | --- | --- | --- | --- | --- | --- | --- | --- | --- | --- |
| HG-CK1 | 91980754 | 94.49 | 5.07 | 89.42 | 44.78 | 44.65 | 44.68 | 44.74 | 57.9 | 31.52 |
| HG-CK 2 | 110912648 | 94.41 | 5.09 | 89.33 | 44.81 | 44.52 | 44.62 | 44.70 | 58.08 | 31.24 |
| HG-CK 3 | 118415530 | 94.50 | 5.21 | 89.29 | 44.72 | 44.57 | 44.61 | 44.68 | 57.79 | 31.50 |
| HG-R1d1 | 119469232 | 95.34 | 5.44 | 89.9 | 45.11 | 44.79 | 44.91 | 44.99 | 58.11 | 31.79 |
| HG-R1d 2 | 115639888 | 94.66 | 5.61 | 89.06 | 44.61 | 44.45 | 44.49 | 44.56 | 57.51 | 31.55 |
| HG-R1d 3 | 92862576 | 94.45 | 5.59 | 88.86 | 44.61 | 44.25 | 44.39 | 44.47 | 57.54 | 31.32 |
| HG-R8d1 | 107205762 | 94.78 | 5.45 | 89.33 | 44.74 | 44.59 | 44.61 | 44.72 | 56.60 | 32.73 |
| HG-R8d2 | 103232946 | 94.93 | 5.44 | 89.5 | 44.83 | 44.66 | 44.69 | 44.80 | 56.68 | 32.82 |
| HG-R8d3 | 87818198 | 94.58 | 5.34 | 89.24 | 44.68 | 44.56 | 44.56 | 44.67 | 56.95 | 32.29 |
| HG-R16d1 | 100957668 | 94.14 | 5.16 | 88.97 | 44.52 | 44.45 | 44.45 | 44.52 | 60.32 | 28.65 |
| HG-R16d2 | 106049098 | 94.16 | 5.12 | 89.04 | 44.75 | 44.29 | 44.47 | 44.57 | 60.37 | 28.67 |
| HG-R16d3 | 143643334 | 93.98 | 5.02 | 88.95 | 44.55 | 44.41 | 44.44 | 44.51 | 60.97 | 27.98 |
| HG-D1d1 | 97088070 | 94.87 | 5.81 | 89.06 | 44.66 | 44.4 | 44.49 | 44.57 | 58.16 | 30.90 |
| HG-D1d2 | 89492142 | 95.12 | 5.90 | 89.22 | 44.74 | 44.48 | 44.57 | 44.65 | 57.68 | 31.54 |
| HG-D1d3 | 125149694 | 95.66 | 6.15 | 89.51 | 44.87 | 44.65 | 44.71 | 44.80 | 58.12 | 31.40 |
| HG-D8d1 | 131607848 | 94.28 | 5.14 | 89.14 | 44.72 | 44.42 | 44.52 | 44.62 | 57.23 | 31.91 |
| HG-D8d2 | 113351812 | 94.74 | 5.50 | 89.21 | 44.86 | 44.35 | 44.54 | 44.67 | 57.27 | 31.94 |
| HG-D8d3 | 117955892 | 94.84 | 5.91 | 88.93 | 44.5 | 44.4 | 44.41 | 44.52 | 57.41 | 31.52 |
| HG-D16d1 | 137432406 | 93.96 | 4.97 | 88.98 | 44.59 | 44.4 | 44.46 | 44.53 | 59.56 | 29.43 |
| HG-D16d2 | 134214208 | 94.18 | 4.85 | 89.33 | 44.77 | 44.56 | 44.62 | 44.70 | 59.83 | 29.49 |
| HG-D16d3 | 156836398 | 93.86 | 4.83 | 89.03 | 44.62 | 44.42 | 44.48 | 44.55 | 59.73 | 29.3 |
| SN-CK1 | 103580818 | 94.85 | 5.72 | 89.13 | 44.67 | 44.46 | 44.51 | 44.62 | 57.12 | 32.01 |
| SN-CK 2 | 99416036 | 95.36 | 5.90 | 89.46 | 44.84 | 44.62 | 44.67 | 44.79 | 56.93 | 32.53 |
| SN-CK 3 | 109203736 | 95.37 | 5.70 | 89.67 | 44.9 | 44.77 | 44.79 | 44.89 | 57.7 | 31.98 |
| SN-R1d1 | 109280530 | 94.68 | 5.57 | 89.11 | 44.77 | 44.35 | 44.52 | 44.60 | 57.18 | 31.93 |
| SN-R1d 2 | 84413250 | 94.58 | 5.85 | 88.73 | 44.42 | 44.31 | 44.33 | 44.40 | 57.20 | 31.53 |
| SN-R1d 3 | 93013924 | 94.25 | 5.71 | 88.54 | 44.33 | 44.21 | 44.24 | 44.30 | 56.82 | 31.71 |
| SN-R8d1 | 117563262 | 95.03 | 5.69 | 89.33 | 44.7 | 44.63 | 44.61 | 44.72 | 57.47 | 31.86 |
| SN-R8d2 | 97765352 | 94.44 | 5.44 | 89.00 | 44.60 | 44.40 | 44.46 | 44.54 | 56.67 | 32.33 |
| SN-R8d3 | 105158986 | 94.52 | 5.45 | 89.07 | 44.66 | 44.41 | 44.47 | 44.59 | 57.2 | 31.87 |
| SN-R16d1 | 102378656 | 94.22 | 4.94 | 89.27 | 44.74 | 44.54 | 44.58 | 44.69 | 58.15 | 31.12 |
| SN-R16d2 | 105358876 | 94.81 | 5.01 | 89.81 | 45.07 | 44.73 | 44.84 | 44.97 | 57.86 | 31.95 |
| SN-R16d3 | 113556996 | 94.33 | 5.01 | 89.32 | 44.72 | 44.60 | 44.61 | 44.71 | 57.78 | 31.54 |
| SN-D1d1 | 94626948 | 95.37 | 5.51 | 89.86 | 44.96 | 44.9 | 44.89 | 44.97 | 57.04 | 32.83 |
| SN-D1d2 | 108098612 | 95.33 | 5.52 | 89.81 | 44.96 | 44.84 | 44.86 | 44.95 | 57.46 | 32.35 |
| SN-D1d3 | 113288258 | 95.11 | 5.26 | 89.85 | 45.03 | 44.82 | 44.88 | 44.97 | 57.94 | 31.91 |
| SN-D8d1 | 105621880 | 94.78 | 5.10 | 89.68 | 44.96 | 44.72 | 44.79 | 44.89 | 57.6 | 32.08 |
| SN-D8d2 | 112517068 | 94.74 | 4.99 | 89.75 | 44.94 | 44.80 | 44.83 | 44.92 | 57.17 | 32.58 |
| SN-D8d3 | 105902248 | 94.59 | 5.02 | 89.57 | 44.88 | 44.68 | 44.73 | 44.83 | 57.37 | 32.19 |
| SN-D16d1 | 116332586 | 94.39 | 4.77 | 89.62 | 44.94 | 44.68 | 44.76 | 44.86 | 57.46 | 32.15 |
| SN-D16d2 | 126656332 | 93.55 | 4.72 | 88.83 | 44.56 | 44.27 | 44.37 | 44.46 | 57.22 | 31.61 |
| SN-D16d3 | 122221430 | 94.73 | 4.93 | 89.80 | 44.96 | 44.84 | 44.86 | 44.95 | 57.66 | 32.15 |

HG, drought-tolerant wheat variety ‘Hengguan35’; SN, drought-sensitive wheat variety ‘Shinong086’. R, rehydrated plants; D, plants under continuous drought stress.

**Table S4.** Quantities of DEGs and differentially-expressed transcription factors upon rehydration and continuous drought stress.

| **Post rehydration** | **Up-regulated genes**  **/ Transcription Factors** | **Down-regulated genes**  **/ Transcription Factors** |
| --- | --- | --- |
| HG-R1d | 6580/2252 | 5493/1948 |
| HG-R8d | 5719/1972 | 8208/3492 |
| HG-R15d | 6137/2269 | 8113/3378 |
| HG-D1d | 7126/2587 | 5466/2086 |
| HG-D8d | 2144/885 | 3973/1606 |
| HG-D15d | 3756/1534 | 7283/2998 |
| SN-R1d | 6804/2762 | 6104/2015 |
| SN-R8d | 5640/2023 | 7069/3045 |
| SN-R15d | 6855/3126 | 7314/2769 |
| SN-D1d | 2529/1005 | 2695/954 |
| SN-D8d | 3066/1205 | 5085/2002 |
| SN-D15d | 4350/1865 | 7128/2716 |

HG, drought-tolerant wheat variety ‘Hengguan35’; SN, drought-sensitive wheat variety ‘Shinong086’. R, rehydrated plants; D, plants under continuous drought stress.
